# Prevalence of Trismus and Its Impact on Oral Health-Related Quality of Life in Patients Treated for Oral Squamous Cell Carcinoma

**DOI:** 10.31557/APJCP.2021.22.8.2437

**Published:** 2021-08

**Authors:** Shailesh M Gondivkar, Amol R Gadbail, Sachin C Sarode, Subhrajit Dasgupta, Balkrishna Sharma, Amol Hedaoo, Aparna Sharma, Gargi S Sarode, Monal Yuwanati, Rima S Gondivkar, Shankargouda Patil, Rahul N Gaikwad

**Affiliations:** 1 *Department of Oral Medicine & Radiology, Government Dental College & Hospital, Nagpur, Maharashtra, India. *; 2 *Department of Dentistry, Indira Gandhi Government Medical College & Hospital, Nagpur, Maharashtra State, India. *; 3 *Department of Oral Pathology & Microbiology, Dr. D.Y. Patil Dental College & Hospital, Dr. D.Y. Patil Vidyapeeth, Pune, Maharashtra State, India. *; 4 *RST Cancer Hospital & Research Centre (Tertiary care cancer centre), Nagpur, Maharashtra State, India. *; 5 *Department of Dentistry, Government Medical College & Hospital, Nagpur, Maharashtra State, India. *; 6 *Department of Oral Pathology and Microbiology. Saveetha Dental College and Hospitals, Saveetha Institute of Medical and Technical Sciences, Saveetha University, Chennai, India. *; 7 *Dental Surgeon, , Aarti regency, Mahalakshmi Nagar, Manewada Road, Nagpur, Maharashtra State, India. *; 8 *Department of Diagnostic Sciences, Division of Oral Pathology, College of Dentistry, Jazan University, Jazan, Kingdom of Saudi Arabia. *; 9 *Department of Community Dentistry and Oral Epidemiology, College of Dentistry, Qassim University, Buraydah, Kingdom of Saudi Arabia. *

**Keywords:** Oral squamous cell carcinoma, trismus, quality of life, oral health, related quality of life, OHIP-14

## Abstract

Oral squamous cell carcinoma, one of the most common malignancies, has a poor prognosis due to impairment in oral functions secondary to treatment. Trismus one of the major causes of impairment of oral function. The present study investigated the prevalence of trismus and its impact on oral health-related quality of life (OHRQoL) in patients treated for oral squamous cell carcinoma (OSCC). The maximum inter-incisal mouth opening of hundred OSCC patients was recorded at post-treatment and 3 months post-treatment. OHRQoL questionnaire (OHIP-14) was intervened to assess the OHRQoL of patients post-treatment and 3 months follow-up, with emphasis on correlation with grades of trismus. The prevalence of trismus was 16% pre-treatment, 72% post-treatment, and 62% at 3 months after treatment. The overall OHIP-14 scores indicated that patients with trismus reported greater impairment of OHRQoL than those without trismus at the end of treatment and 3 months follow-up. At the end of treatment, patients with severe trismus demonstrated a higher mean OHIP-14 score (23.47 ±3.34) than those with moderate (17.72 ±2.83) and mild trismus (12.66 ±3.84) with statistically significant differences (p<0.001). Equivalent results were obtained at 3 months follow-up period. Patients with trismus suffer greater impairment of OHRQoL. The findings demand the need of identifying risk factors for developing trismus and early institution of newer/modified treatment approaches for better OHRQoL in OSCC survivors.

## Introduction

Oral squamous cell carcinoma (OSCC) is the sixth most common malignancy in the world and is highly prevalent in South-Central Asia (Feller and Lemmer, 2012; Hernández-Guerrero et al., 2013). In India, OSCC is the most common malignant neoplasm in males and third most common in females. Over 1,000,000 new cases of OSCC are registered every year in India (Warnakulasuriya, 2009). Despite of several advancement in treatment options over the years, the 5-year survival rate has not significantly improved and is around 40-50% in most of the countries (Markopoulos, 2012; Shenoi et al., 2012). The curative management of OSCC not only affects cosmetic appearance but also debilitate the integrity and vital functions of oral cavity such as speech, taste, chewing and swallowing. These oral dysfunctions adversely affect health-related quality of life (HRQoL) and hence should be addressed with utmost priority (Epstein et al., 2001; Kreeft et al., 2009). Owing to complex tri-dimensional anatomy of the mouth and proximity to vital structures, OSCC patients are considered different than those with other head and neck (H&N) cancers and use of site-specific analysis of outcomes has been recommended in the literature (Chandu et al., 2006; Barrios et al., 2015).

Trismus is defined as an inability to open the mouth or difficulty in mouth opening mostly secondary to spasm of the muscles of mastication (Melchers et al, 2009; Bensadoun et al, 2010). Healthy individuals usually have maximal inter-incisal mouth opening (MIO) in the range of 36-55 mm and MIO of ≤ 35 mm is regarded as trismus (Dijkstra et al., 2006). Trismus in OSCC patients can be caused by various factors such as infiltration of tumour in the muscles of mastication and temporomandibular joint, radiation-induced fibrosis, post-surgical scarring (Tanaka, 1993; Stubblefield et al., 2010), or associated pre-existing oral submucous fibrosis (Gadbail et al., 2017). The literature reported variation in the incidences of trismus in treated H&N cancer patients with a range of 28% to 79% (Johnson et al., 2010; Lee et al., 2012; Pauli et al., 2013; Wetzels et al., 2014; Steiner et al., 2015). This variation could be attributed to non-homogeneity of inclusion criteria, cut-off values of trismus and follow-up periods. The trismus in these patients results in persisting great difficulties in routine day-to-day activities including chewing, drinking, speaking, maintaining oral hygiene and receiving dental interventions, thereby deteriorates the oral health-related quality of life (OHRQoL) and over the time leads to impairment of different aspects of HRQoL (Lee et al., 2012).

Although trismus has been recognized to have large impacts on HRQoL in patients treated for H & N cancer, there is still lack of knowledge about its impact on OHRQoL in OSCC treated patients. Looking at the continuously increasing number of OSCC cases globally and being a neglected part, the present study was designed to estimate the prevalence of trismus and to analyze its impacts on OHRQoL in patients treated for OSCC.

*Objective*

Primary objective: To determine the effect of trismus on OHQoL in OSCC-treated patients.

## Materials and Methods

*Study design and patients*

The present cross-sectional, observational, prospective study was conducted in all OSCC patients treated at tertiary care cancer hospital in India during a period of June 2019 to May 2020. Eligibility criteria were histopathological confirmed diagnosis of OSSC; treated with primary surgery, with or without adjuvant radiotherapy (RT); cancer-free survival for > 3 months; all age-gender. Patients were excluded if they: have terminal stage OSCC; with systemic disease; received pre-treatment chemo or radiotherapy; unable to complete questioner. Informed consent was collected from all enrolled patient prior to data collection. The study protocol was approved by the Institutional Ethics Committee of Cancer Relief Society’s RST Regional Cancer Hospital, Nagpur, India, on 20th June 2019 (approval no. RST/RCH: 444).

*Data collection and QoL instrument used*

Baseline characteristics on patents age, gender, habit, tumor site, TNM stage, histopathological variant, treatment (surgical treatment, radiation dosages and CT cycles), comorbidity. Inter incisal distance (MIO) and OH-QoL was measured preoperatively, post-operatively and 3 months after the treatment. 

MIO was measured using digital caliper in millimeter. (Dijkstra et al., 2006) Trismus was classified based on criteria as mild (31-35 mm), moderate (26-30 mm) and severe (< 25 mm) (Thomas et al., 1988; Martins et al., 2020). In edentulous patients, distance between alveolus ridge was measured. 

OHRQoL was measured using validated 14 item short version of OHIP- Hindi language (OHIP-14). The OHIP-14 includes seven dimensions of impact: functional limitation, physical pain, psychological discomfort, physical disability, psychological disability, social disability and handicap. Trained specialists recorded response from the patients on a 5-point Likert scale (ranging from 0 to 4): never (0), hardly ever (1), occasionally (2), fairly often (3), and very often (4) (Slade et al, 2005) Additive method was used to obtain overall OHRQoL (range 0-56). A higher total score indicates a poor OHRQoL. 

*Statistical analysis*

Mean score for MIO and OHIP14 was calculated The Statistical Package for the Social Sciences (SPSS), version 17.0 was used for statistical analysis. Patients characteristics were presented using descriptive statistics as number, percentage, mean and standard deviation. To evaluate the associations between the OHIP (dependent variable) and MIO, histopathology, treatment, tumor site, TNM stage (independent variables), multiple linear regression analysis was performed. In addition, to determine the difference in between MIO and OHIP score, postoperatively and at 3 months follow-up, paired t-test was conducted. 

## Results

*Demographic characteristics*

Hundred and fifteen patients with OSCC were admitted between June 2019 between May 2020 to the tertiary Cancer care Hospital: 15 were excluded, and 100 met the inclusion criteria. Twelve patients were non-eligible for the reasons including: six patients denied for participation, four secondary to poor general health and two for unspecified reasons. Three patients died prior to follow-up at 3 months post-treatment.

Characteristics of OSCC patients are reported in Table 1. Mean age of the patients was 47.2 ± 12.11 (median 45; range 20-80) years and males (77%) comprised more than two third of the study participants. The most frequent site of OSCC was gingivo-buccal sulcus (42%) followed by buccal mucosa (23%). The 48 % OSCC patient were in stage IV. 47 patients were treated with surgery alone whereas 22 patient with additional RT. Rest of the patients with surgery, adjuvant RT concurrent chemoradiation (n=31). 

The prevalence of trismus among the OSCC treated patients was 72 %. The mean MIO at posteratively and three follow-up was 28.3 ± 7.6 and 31.5 ± 8.2 mm (Table 2). 72 patients had trismus after treatment whereas 62 patients had trismus after 3 months. Out of 72 patient with trismus postoperatively, 11, 15, and 46 had mild, moderate, and severe, respectively. After 3 months follow-up, 8, 21, and 33 had mild, moderate, and severe trismus, respectively. The MIO after 3 months shows significant improvement with mean difference 3.23 mm (95 %CI, (3.45-3.01), P < .001,) (Table 3). The mean MIO in OSCC patient treated with surgery, Surgery and RT, and surgery, RT and chemotherapy was Table 4. The OSCC patient have undergone the combined treatment had less MIO or more trismus than patient treated with trismus or surgery plus radiotherapy suggestive of influence of treatment in development of trismus in our study patients. 

**Figure 1:**
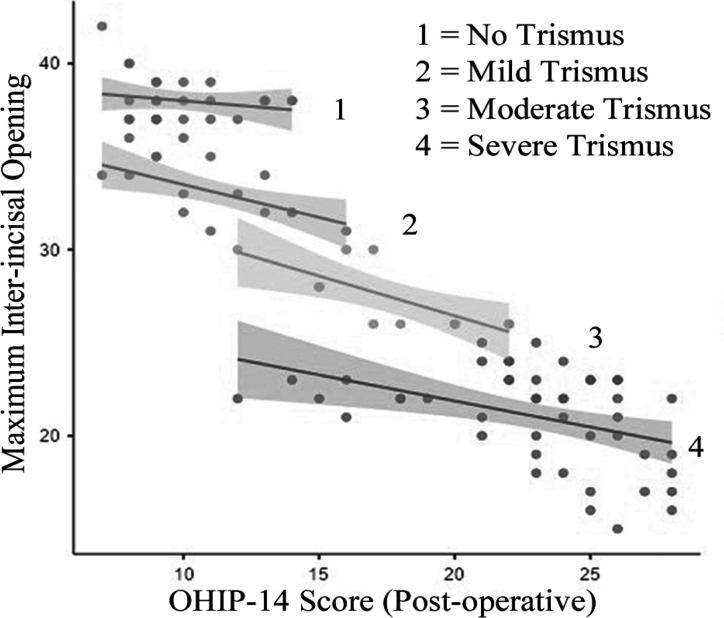
Correlation of OHIP Score and Mouth Opening (Post-Operative)

**Figure 2:**
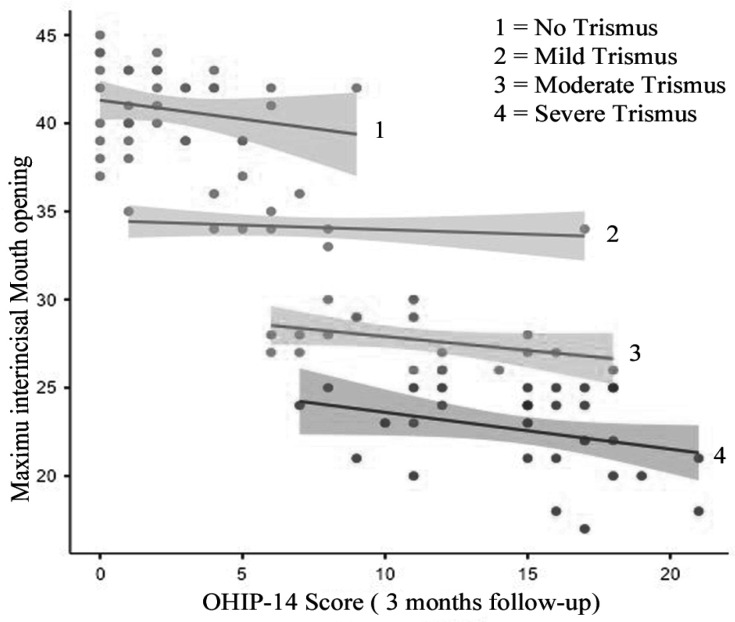
Correlation of OHIP Score and Mouth Opening (3 Months Follow-up)

The validated Hindi version of OHIP-14 questionnaire used. Total OHIP score was calculated using additive method with range 0-56. Postoperatively the mean OHIP score was 17 ± 6.7 whereas it was improved after 3 months to 8.6 ± 6.2 with mean difference of 8.37 (95 % CI (7.92-8.82), P= < .001). The trismus had a significant negative correlation with OHIP score with 1 mm increase trismus causing 0.4741 increase OHIP score. (R^2^ .809, P= <.001) (Figure 1 and Figure 2). In addition, those with severe trismus and moderate trismus posteratively had higher OHIP score as compared to those without trismus or mild trismus. 

We also evaluated the age; gender, site, histology, TNM grade, and treatment variables influence postoperative and three-month follow-up OHIP score (Table 5 and Table 6). Except treatment, no other variable were able to predict the higher OHIP score postoperatively and after 3 months. Those treated with combined modalities and IV TNM stage had a significant impact on OHIP score than patients treated with surgery or surgery with radiotherapy. Combined modality treated caused 0.94 increase in OHIP score. 

**Table 1:** Characteristics of OSCC Patients

	(N=100) %
Age - Age Cat	
<45	54 (54.0%)
>45	46 (46.0%)
Gender	
Male	77 (77.0%)
Female	23 (23.0%)
Histology Variants	
WDSCC	37 (37.0%)
MDSCC	55 (55.0%)
PDSCC	8 (8.0%)
Treatment	
Surgery	47 (47.0%)
Surgery + Radio	22 (22.0%)
Surgery + Radio(chemo)	31 (31.0%)
TNM stage	
I	16 (16.0%)
II	17 (17.0%)
III	19 (19.0%)
IV	48 (48.0%)
Site-Cat	
Labial Mucosa	5 (5.0%)
Buccal mucosa	24 (24.0%)
GB sulcus	43 (43.0%)
Palate	4 (4.0%)
Tongue	14 (14.0%)
Retromolar	9 (9.0%)
Floor of mouth	1 (1.0%)

**Table 2:** Levels of Trismus among Different Variables

	No trismus (N=28)	Mild (N=15)	Moderate (N=11)	Severe (N=46)	Total (N=100)	p value
OHIP *						< 0.001^1^
Mean (SD)	10.0 (2.0)	11.4 (2.6)	17.6 (3.1)	23.0 (3.9)	17.0 (6.7)	
Range	7.0 - 14.0	7.0 - 16.0	12.0 - 22.0	12.0 - 28.0	7.0 - 28.0	
MIO *						< 0.001^1^
Mean (SD)	38.0 (1.3)	33.0 (1.4)	27.5 (1.8)	21.0 (2.5)	28.3 (7.6)	
Range	36.0 - 42.0	31.0 - 35.0	26.0 - 30.0	15.0 - 25.0	15.0 - 42.0	
MIO **						< 0.001^1^
Mean (SD)	41.8 (1.6)	36.7 (2.1)	30.4 (2.2)	23.8 (2.8)	31.5 (8.2)	
Range	39.0 - 45.0	34.0 - 40.0	28.0 - 34.0	17.0 - 29.0	17.0 - 45.0	
OHIP **						< 0.001^1^
Mean (SD)	2.3 (2.2)	3.3 (2.7)	10.3 (3.3)	13.8 (4.0)	8.6 (6.2)	
Range	0.0 - 9.0	0.0 - 8.0	6.0 - 17.0	6.0 - 21.0	0.0 - 21.0	
Gender						0.013^2^
Male	25.0 (89.3%)	12.0 (80.0%)	11.0 (100.0%)	29.0 (63.0%)	77.0 (77.0%)	
Female	3.0 (10.7%)	3.0 (20.0%)	0.0 (0.0%)	17.0 (37.0%)	23.0 (23.0%)	
Histology Variant						< 0.001^2^
WDSCC	25.0 (89.3%)	8.0 (53.3%)	0.0 (0.0%)	4.0 (8.7%)	37.0 (37.0%)	
MDSCC	3.0 (10.7%)	7.0 (46.7%)	9.0 (81.8%)	36.0 (78.3%)	55.0 (55.0%)	
PDSCC	0.0 (0.0%)	0.0 (0.0%)	2.0 (18.2%)	6.0 (13.0%)	8.0 (8.0%)	
Site						< 0.001^2^
Labial Mucosa	5.0 (17.9%)	0.0 (0.0%)	0.0 (0.0%)	0.0 (0.0%)	5.0 (5.0%)	
Buccal mucosa	9.0 (32.1%)	3.0 (20.0%)	3.0 (27.3%)	9.0 (19.6%)	24.0 (24.0%)	
GB sulcus	13.0 (46.4%)	9.0 (60.0%)	8.0 (72.7%)	13.0 (28.3%)	43.0 (43.0%)	
Palate	1.0 (3.6%)	1.0 (6.7%)	0.0 (0.0%)	2.0 (4.3%)	4.0 (4.0%)	
Tongue	0.0 (0.0%)	1.0 (6.7%)	0.0 (0.0%)	13.0 (28.3%)	14.0 (14.0%)	
Retromolar	0.0 (0.0%)	0.0 (0.0%)	0.0 (0.0%)	9.0 (19.6%)	9.0 (9.0%)	
Floor of mouth	0.0 (0.0%)	1.0 (6.7%)	0.0 (0.0%)	0.0 (0.0%)	1.0 (1.0%)	
TNM stage						< 0.001^2^
I	16.0 (57.1%)	0.0 (0.0%)	0.0 (0.0%)	0.0 (0.0%)	16.0 (16.0%)	
II	9.0 (32.1%)	6.0 (40.0%)	0.0 (0.0%)	2.0 (4.3%)	17.0 (17.0%)	
III	3.0 (10.7%)	9.0 (60.0%)	0.0 (0.0%)	7.0 (15.2%)	19.0 (19.0%)	
IV	0.0 (0.0%)	0.0 (0.0%)	11.0 (100.0%)	37.0 (80.4%)	48.0 (48.0%)	
Treatment						< 0.001^2^
Surgery	28.0 (100.0%)	15.0 (100.0%)	0.0 (0.0%)	4.0 (8.7%)	47.0 (47.0%)	
Surgery + Radio	0.0 (0.0%)	0.0 (0.0%)	10.0 (90.9%)	12.0 (26.1%)	22.0 (22.0%)	
Surgery + Radio(chemo)	0.0 (0.0%)	0.0 (0.0%)	1.0 (9.1%)	30.0 (65.2%)	31.0 (31.0%)	

*, Postoperative, ** 3 months follow-up; ^1^, Linear Model ANOVA; ^2^, Pearson's Chi-squared test

**Table 3:** Comparison of MIO and OHIP Postoperatively and after 3 Months

Paired Samples T-Test								95% Confidence Interval	
			statistic	df	p	Mean difference	SE difference	Lower	Upper
MIO-po	MIO-3f	Student's t	-28.7	99	<0.001	-3.23	0.113	-3.45	-3.01
OHIP-po	OHIP-3f	Student's t	36.9	99	<0.001	8.37	0.227	7.92	8.82

**Table 4:** Mean MIO and OHIP Score in OSCC Treated Patients

	Surgery (N=47)	Surgery + Radio (N=22)	Surgery + Radio(chemo) (N=31)	Total (N=100)	p value
MIO-po					< 0.001^2^
Mean (SD)	35.1 (4.7)	24.1 (3.8)	21.0 (2.9)	28.3 (7.6)	
Range	22.0 - 42.0	17.0 - 30.0	15.0 - 26.0	15.0 - 42.0	
OHIP-po					< 0.001^2^
Mean (SD)	10.9 (2.9)	19.5 (3.8)	24.5 (2.5)	17.0 (6.7)	
Range	7.0 - 22.0	12.0 - 27.0	18.0 - 28.0	7.0 - 28.0	
MIO-3f					< 0.0012
Mean (SD)	38.9 (4.7)	27.0 (4.0)	23.5 (3.0)	31.5 (8.2)	
Range	25.0 - 45.0	20.0 - 34.0	17.0 - 29.0	17.0 - 45.0	
OHIP-3f					< 0.001^2^
Mean (SD)	3.1 (2.8)	9.9 (2.4)	16.1 (2.3)	8.6 (6.2)	
Range	0.0 - 12.0	6.0 - 17.0	11.0 - 21.0	0.0 - 21.0	

^1^, Pearson's Chi-squared test; ^2^, Linear Model ANOVA

**Table 5:** Multiple Linear Regression Analysis for OHIP, Trismus and Characteristics of OSCC Patient (Postoperative Evaluation)

			95% Confidence Interval				Stand. Estimate	95% Confidence Interval	
Predictor	Estimate	SE	Lower	Upper	t	p		Lower	Upper
Intercept ᵃ	20.0943	7.213	5.735	34.453	2.786	0.007			
Mouth Opening	-0.247	0.188	-0.622	0.128	-1.3119	0.193	-0.28088	-0.7071	0.1454
Histology Variant:									
MDSCC – WDSCC	1.8124	1.471	-1.117	4.742	1.2318	0.222	0.26949	-0.1661	0.705
PDSCC – WDSCC	-0.3226	1.782	-3.87	3.225	-0.181	0.857	-0.04797	-0.5755	0.4795
Site-Cat:									
Labial Mucosa – Buccal mucosa	-1.8698	1.237	-4.333	0.593	-1.5112	0.135	-0.27801	-0.6443	0.0882
GB sulcus – Buccal mucosa	0.0336	0.68	-1.319	1.387	0.0495	0.961	0.005	-0.1962	0.2062
Palate – Buccal mucosa	-1.2579	1.293	-3.832	1.316	-0.9728	0.334	-0.18703	-0.5698	0.1957
Tongue – Buccal mucosa	0.3125	1.033	-1.744	2.368	0.3026	0.763	0.04646	-0.2592	0.3522
Retromolar – Buccal mucosa	1.0165	1.292	-1.555	3.588	0.787	0.434	0.15114	-0.2312	0.5335
Floor of mouth – Buccal mucosa	0.581	2.664	-4.722	5.884	0.2181	0.828	0.08638	-0.7021	0.8749
TNM stage:									
II – I	-0.7184	0.931	-2.572	1.135	-0.7716	0.443	-0.10682	-0.3824	0.1688
III – I	-2.3795	1.711	-5.785	1.027	-1.3908	0.168	-0.3538	-0.8602	0.1526
IV – I	-0.5055	2.451	-5.386	4.375	-0.2062	0.837	-0.07517	-0.8008	0.6504
Treatment:									
Surgery + Radio – Surgery	3.8816	1.88	0.139	7.624	2.0648	0.042	0.57715	0.0207	1.1336
Surgery + Radio(chemo) – Surgery	6.5671	1.846	2.891	10.243	3.5569	< .001	0.97645	0.4299	1.523
Gender:									
Female – Male	-0.8462	0.644	-2.128	0.435	-1.3146	0.192	-0.12582	-0.3164	0.0647
Trismus									
Mild – No trismus	-0.1503	1.272	-2.683	2.382	-0.1182	0.906	-0.02235	-0.3989	0.3542
Moderate – No trismus	-4.83	2.671	-10.147	0.487	-1.8085	0.074	-0.71816	-1.5087	0.0724
Severe – No trismus	-3.834	2.884	-9.575	1.908	-1.3294	0.188	-0.57006	-1.4237	0.2836

ᵃ, Represents reference level

## Discussion

The presented evaluated trismus and its impact on OHRQoL in patients treated for OSCC. Out results showed that trismus could influence the OHRQoL in OSCC patient after treatment. Past studies have witnessed long term impacts of trismus on HRQoL in H&N cancer treated patients (Dijkstra et al., 2006; Lee et al., 2012). However, due to limited literature on OHRQoL, there was a dire need of identifying the prevalence of trismus and its impact on OHRQoL in OSCC patients. In the present study, we have used criteria for trismus put forward by Dijkstra et al., (2006) as it is widely accepted and considered as a gold standard (Louise Kent et al., 2008; Scott et al., 2011; Pauli et al., 2013). At the end of treatment, a reduction in MIO was reported in all patients, which is in accordance with the previous studies (Wetzels et al., 2014; Galitis et al., 2017). In the present study, the prevalence of trismus was highest (72%) at post-treatment, and then declined to 62% at 3 months follow-up. Recent studies documented post-treatment trismus incidence rates of 28% and 44% in H&N cancer patients (Wetzels et al., 2014; Steiner et al., 2015). However, 33%, 42% and 79% post-treatment incidence rates were reported by other past studies (Johnson et al., 2010; Lee et al., 2012; Pauli et al., 2013). This indicates that the reported incidences of trismus in patients with H&N cancer exhibits varied results. The prevalence of trismus reported in the present study is within the range reported in the literature.

OSCC patient in this study were treated with different treatment modalities. Overall, the patients treated for OSCC with various treatment modalities showed increased prevalence of trismus and deteriorated OHRQoL. However, combined treatment involving radiotherapy and chemotherapy is risk for development of trismus, thereby can affect OHQoL. In particular, those who received post-operative RT combined with CT demonstrated significantly increased trismus and worse OHRQoL. These patients due to radiation develops oral symptoms such as swallowing problems, taste alterations, sticky saliva, dry mouth, coarseness, and dental problems.(Galitis et al., 2017) Due these symptoms interfere with daily activities resulting in poor OHQoL. Hence, utmost attention should be given in future studies to improve OHRQoL in such patients. 

The resultant improvement in mean MIO at 3 months follow-up after oncological treatment was noticed to be highest in patients treated by surgery alone as compared to those who received primary surgery and adjuvant RT and/or CRT. Suppression of tissue regenerative and repair capacity due to radiotherapy and chemotherapy could be the reason for delayed or negative response in improvement of mouth opening. Loorents et al. Loorents et al., (2016) reported gradual improvement in mouth opening at 3 months to one year after completed RT in patients with H&N cancer. However, et al., (2014) did not observed any improvement of MIO one year after post-operative RT. The observations of the present study supports the noted declined prevalence rate of trismus from 72% at post-treatment to 62% at 3 months follow-up. These results could be attributed to the fact that 47% patients were treated by surgery without adjuvant RT or CRT in the present study. Moreover, few patients had mild trismus at the end of treatment that might have shown improvement in MIO at 3 months follow-up. However, reasons such as buccal mucosa as the second most common site, early stage, young age etc. cannot be ruled out as possible factor for post-operative improvement of in trismus. All these factors together might have contributed to early recovery of MIO in some patients. However, to get better insight into this perspective future focused studies are warranted.

Numerous QoL instruments are available in the literature to assess HRQoL in OSCC patients (Chandu et al., 2006; Carranza et al., 2008). However, because of the functional and anatomy distinctness of the oral cavity, it is advisable to use specific OHRQoL tools, which are more sensitive to evaluate impacts of oral condition on day-to-day activities. In the present study, OHIP-14 was used to assess OHRQoL due to its worldwide acceptability, reliability, sensitivity to change and adequate cross-cultural consistency (Montero-Martin et al., 2009; Rana et al., 2015). 

The results of the present study demonstrated higher overall OHIP-14 scores indicating significantly worse OHRQoL in patients with trismus than non-trismus patients at the end of treatment and at 3 months follow-up. This is in agreement with the two recent studies, which has used same instrument for OHRQoL assessment (Barrios et al., 2015; Indrapriyadharshini et al., 2017). Majority of the patients reported difficulty in eating that could be related to the previous reports of difficulty in chewing and swallowing in patients treated for oral and oropharygeal cancer (Biazevic et al., 2010; Dwivedi et al., 2012) We believe that the observed impairments in the present study are partially in accordance with the prevalence of trismus. For better understanding of this aspect, we have also assessed and compared the effects of trismus on OHRQoL as per the degree of severity of the trismus. It was observed that a significant number of patients with severe trismus exhibited higher overall OHIP-14 scores than those with moderate and mild trismus in the decreasing order, indicating significantly worse OHRQoL in patients with severe trismus. These observations related to trismus and impacts on OHRQoL are similar at end of treatment and 3-months follow-up category. Past studies advocated trismus as an important and independent risk factor for impairment of HRQoL and recommended necessity of support to oral structures in order to prevent/manage trismus in H&N cancer patients (Lee et al., 2012; Pauli et al., 2013; Wetzels et al., 2014; Steiner et al., 2015). In agreement with this, present study demonstrated that OHRQoL corresponds with the degree of trismus, and clinical judgment and early institution of preventive rehabilitation approaches would help in limiting the post-treatment trismus and improving the OHRQoL in OSCC patients.

The main strength of the present study is the quantification of MIO in millimetres rather than simple binary measurements (presence and absence). This allowed us to define patients as per degree of trismus and to perform a more comprehensive analysis. In addition, due to prospective nature of the study, a stable analysis of MIO and OHRQoL changes over the period was performed. In the present study, we have eliminated the important confounding factor related to heterogeneity in the type of H&N cancer by restricting inclusion of patients to only OSCC. 

Due to generic nature of the OHRQoL instrument, the possibility of impact of other oral conditions that are present concomitantly with the OSCC (before and after treatment) cannot be ruled out. Therefore, OHRQoL could have assessed using OC-specific OHRQoL measure and should be included in future studies. However, as generic measures are conceptually broader, it covers larger aspects of patients’ health. In the present study, no precise data were available regarding use of physiotherapy regimens in patients with trismus. Therefore, the present study could not evaluate the effects of physiotherapy on MIO. In addition, the follow-up included in the present study is short which did not allow us to assess further progression or remission of trismus and long-term impacts of trismus on OHRQoL. With the period of molecular remodelling in repair and regeneration in mind, we believe that 3-month period is ideal to judge the future impact of trismus. However, future prospective studies with longer duration with multiple time point analysis of parameters are warranted to have better insight. Few patients had trismus prior to start of oncological treatment. This might have contributed a bit too increased prevalence of post-treatment trismus noted in the present study. The findings of the present study should be interpreted with caution as the results obtained might not be generalizable to other parts of the world where tongue or floor of mouth are common sites for OSCC and trismus can be rarely present. 

In conclusion, present study extends previous research and helps to fill the knowledge gap about the trismus prevalence and its impact on OHRQoL in patients treated for OSCC with various treatment modalities. The higher prevalence of post-treatment trismus and lower remission at 3 months follow-up was evident in patients treated with post-operative RT/CRT. The noteworthy finding of present study was that with the advancing degree of trismus we observed an increase in worsening of the OHRQoL. The observations of this study demands that clinicians and caregivers should identify OSCC patients who are at high risk for developing trismus while planning management strategies for them. To authenticate the findings, multi-centric cohort studies on larger sample size and long duration follow-up are warranted. Moreover, future prospective studies using physiotherapy regimes and newer/modified treatment approaches for prevention/treatment of trismus in order to improve OHRQoL of OSCC survivors are recommended. 

## Author Contribution Statement

The Study was designed and conceived by SG, AG, SS and SD. BS, AM, RG and AS analyzed the data. GS and MY wrote the manuscript. SP and RG guided the execution of the study and revised the paper. All authors read and approved the final version of this manuscript.

## References

[B1] Barrios R, Bravo M, Gil-Montoya JA (2015). Oral and general health-related quality of life in patients treated for oral cancer compared to control group. Health Qual Life Outcomes.

[B2] Bensadoun RJ, Riesenbeck D, Lockhart PB (2010). A systematic review of trismus induced by cancer therapies in head and neck cancer patients. Support Care Cancer.

[B3] Biazevic MGH, Antunes JLF, Togni J (2010). Survival and quality of life of patients with oral and oropharyngeal cancer at 1-year follow-up of tumor resection. J Appl Oral Sci.

[B4] Carranza ET, Cossío PI, Guisado JMH, Aumente EH, Pérez JLG (2008). Assessment of quality of life in oral cancer. Med Oral Patol Oral Cir Bucal.

[B5] Chandu A, Smith ACH, Rogers SN (2006). Health-related quality of life in oral cancer: A review. J Oral Maxillofac Surg.

[B6] Dijkstra PU, Huisman PM, Roodenburg JLN (2006). Criteria for trismus in head and neck oncology. Int J Oral Maxillofac Surg.

[B7] Dwivedi RC, Chisholm EJ, Khan AS (2012). An exploratory study of the inXuence of clinico-demographic variables on swallowing and swallowing-related quality of life in a cohort of oral and oropharyngeal cancer patients treated with primary surgery. Eur Arch Oto-Rhino-Laryngol.

[B8] Epstein JB, Robertson M, Emerton S, Phillips N, Stevenson-Moore P (2001). Quality of life and oral function in patients treated with radiation therapy for head and neck cancer. Head Neck.

[B9] Feller L, Lemmer J (2012). Oral squamous cell carcinoma: Epidemiology, Clinical Presentation and Treatment. J Cancer Ther.

[B10] Gadbail AR, Chaudhary M, Gawande M (2017). Oral squamous cell carcinoma in the background of oral submucous fibrosis is a distinct clinicopathological entity with better prognosis. J Oral Pathol Med.

[B11] Galitis E, Droukas V, Tzakis M (2017). Trismus and reduced quality of life in patients with oral squamous cell carcinoma, who received post-operative radiotherapy alone or combined with chemotherapy. Forum Clin Oncol.

[B12] Hernández-Guerrero JC, Jacinto-Alemán LF, Jiménez-Farfán MD (2013). Prevalence trends of oral squamous cell carcinoma Mexico City’s general hospital experience. Med Oral Patol Oral Cir Bucal.

[B13] Indrapriyadharshini K, Madankumar PD, Karthikeyan GR (2017). Oral health-related quality of life in patients treated for oral malignancy at Kanchipuram district, India: A cross-sectional study. Indian J Cancer.

[B14] Johnson J, Van as-Brooks CJ, Fagerberg-Mohlin B, Finizia C (2010). Trismus in head and neck cancer patients in sweden: Incidence and risk factors. Med Sci Monit.

[B15] Kreeft AM, Van Der Molen L, Hilgers FJ, Balm AJ (2009). Speech and swallowing after surgical treatment of advanced oral and oropharyngeal carcinoma: A systematic review of the literature. Eur Arch Oto-Rhino-Laryngol.

[B16] Lee R, Slevin N, Musgrove B, Swindell R, Molassiotis A (2012). Prediction of post-treatment trismus in head and neck cancer patients. Br J Oral Maxillofac Surg.

[B17] Loorents V, Rosell J, Salgado Willner H, Börjeson S (2016). Health-related quality of life up to 1 year after radiotherapy in patients with head and neck cancer (HNC). Springerplus.

[B18] Louise Kent M, Brennan MT, Noll JL (2008). Radiation-induced trismus in head and neck cancer patients. Support Care Cancer.

[B19] Markopoulos AK (2012). Current aspects on oral squamous cell carcinoma. Open Dent J.

[B20] Martins CA, Goldenberg DC, Narikawa R, Kowalski LP (2020). Trismus and oral health conditions during diagnosis of malignant oral neoplasms. Braz J Otorhinolaryngol.

[B21] Melchers LJ, Van Weert E, Beurskens CHG (2009). Exercise adherence in patients with trismus due to head and neck oncology: a qualitative study into the use of the Therabite®. Int J Oral Maxillofac Surg.

[B22] Montero-Martin J, Bravo-Pérez M, Albaladejo-Martínez A, Hernández-Martin LA, Rosel-Gallardo EM (2009). Validation the Oral Health Impact Profile (OHIP-14sp) for adults in Spain. Med Oral Patol Oral Cir Bucal.

[B23] Pauli N, Johnson J, Finizia C, Andréll P (2013). The incidence of trismus and long-term impact on health-related quality of life in patients with head and neck cancer. Acta Oncol (Madr).

[B24] Rana M, Kanatas A, Herzberg PY, Gellrich NC, Rana M (2015). Relevance of psychosocial factors to quality of life in oral cancer and oral lichen planus: a prospective comparative study. Br J Oral Maxillofac Surg.

[B25] Scott B, D’Souza J, Perinparajah N, Lowe D, Rogers SN (2011). Longitudinal evaluation of restricted mouth opening (trismus) in patients following primary surgery for oral and oropharyngeal squamous cell carcinoma. Br J Oral Maxillofac Surg.

[B26] Shenoi R, Devrukhkar V, Chaudhuri (2012). Demographic and clinical profile of oral squamous cell carcinoma patients: A retrospective study. In: Indian Journal of Cancer. Indian J Cancer.

[B27] Slade GD, Nuttall N, Sanders AE (2005). Impacts of oral disorders in the United Kingdom and Australia. Br Dent J.

[B28] Steiner F, Evans J, Marsh R (2015). Mouth opening and trismus in patients undergoing curative treatment for head and neck cancer. Int J Oral Maxillofac Surg.

[B29] Stubblefield MD, Manfield L, Riedel ER (2010). A preliminary report on the efficacy of a dynamic jaw opening device (dynasplint trismus system) as part of the multimodal treatment of trismus in patients with head and neck cancer. Arch Phys Med Rehabil.

[B30] Tanaka T (1993). Trismus in patients with malignant tumours in the head and neck. J Laryngol Otol.

[B31] Thomas F, Ozanne F, Mamelle G, Wibault P, Eschwege F (1988). Radiotherapy alone for oropharyngeal carcinomas: the role of fraction size (2 Gy vs 2 5 Gy) on local control and early and late complications. Int J Radiat Oncol Biol Phys.

[B32] Warnakulasuriya S (2009). Global epidemiology of oral and oropharyngeal cancer. Oral Oncol.

[B33] Wetzels J-WGH, Merkx MAW, de Haan AFJ, Koole R, Speksnijder CM (2014). Maximum mouth opening and trismus in 143 patients treated for oral cancer: a 1-year prospective study. Head Neck.

